# Upfront intensive chemo-immunotherapy with autograft in 199 adult mantle cell lymphoma patients: prolonged survival and cure potentiality at long term

**DOI:** 10.1038/s41409-021-01391-x

**Published:** 2021-07-07

**Authors:** Sergio Cortelazzo, Michael Mian, Andrea Evangelista, Liliana Devizzi, Paolo Corradini, Michele Magni, Marco Ladetto, Simone Ferrero, Andrea Rossi, Anna Maria Barbui, Caterina Patti, Alessandro Costa, Umberto Vitolo, Annalisa Chiappella, Fabio Benedetti, Andrés J. M. Ferreri, Paolo Nicoli, Luigi Rigacci, Claudia Castellino, Alessandro M. Gianni, Alessandro Rambaldi, Corrado Tarella

**Affiliations:** 1 Oncology Unit, Clinica Humanitas/Gavazzeni, Bergamo, Italy; 2 Division of Hematology and BMT, Ospedale Centrale di Bolzano, Bolzano, Italy; 3 Faculty of Medicine of the Riga Stradins University (RSU), Riga, Lativa Italy; 4 Unità di Epidemiologia Clinica e Valutativa AOU Città della Salute e della Scienza di Torino e CPO Piemonte, Torino, Italy; 5 Division of Hematology and Stem Cell Transplantation, Fondazione IRCCS Istituto Nazionale dei Tumori, Milano, Italy; 6 Department of Onco-Hematology, University of Milan, Milano, Italy; 7 Oncologia Medica 1, Fondazione IRCCS Istituto Nazionale dei Tumori, Milano, Italy; 8 Division of Hematology, AO SS Antonio e Biagio e Cesare Arrigo, Alessandria, Italy; 9 Department of Molecular Biotechnologies and Health Sciences, University of Torino, Italy/Hematology 1, “AOU Città della Salute e della Scienza di Torino”, Torino, Italy; 10 UOC Ematologia, ASST Papa Giovanni XXIII, Bergamo, Italy; 11 Hematology Unit, Ospedale V. Cervello, Palermo, Italy; 12 Multidisciplinary Outpatient Oncology Clinic, Candiolo Cancer Institute, FPO-IRCCS, Candiolo, Torino, Italy; 13 Department of Medicine, Section of Hematology, University of Verona, Verona, Italy; 14 Lymphoma Unit, Department of Onco-Hematology, IRCCS San Raffaele Scientific Institute, Milano, Italy; 15 Hematology Unit, University of Turin, Orbassano, Italy; 16 U.O. di Ematologia e Trapianti di cellule Staminali. A.O.S. S. Camillo-Forlanini, Roma, Italy; 17 Hematology Unit, Ospedale S. Croce e Carle, Cuneo, Italy; 18 Onco-Hematology Unit, European Institute of Oncology IRCCS, Milano, Italy; 19 Department of Health Sciences, University of Milan, Milano, Italy

**Keywords:** Stem-cell research, Stem-cell therapies, Risk factors

## To the Editor:

Mantle cell lymphoma (MCL) is a rare disease with an aggressive clinical course in most patients. This resulted in a median survival of MCL of only 3–5 years [1]. Intensive first-line chemo-immunotherapy with autograft has improved the clinical outcome of MCL. However, the clinical course remains heterogeneous and can only be partially predicted with internationally validated prognostic scores, such as the MCL international prognostic index (MIPI) [2]. Moreover, the long-term outcome remains a matter of concern in MCL patients [3–5]. To investigate long-term outcomes in a sufficiently large patient population, we performed a retrospective cohort analysis of 199 adult MCL patients treated at 13 Italian cancer centres, with the intensive rituximab and cytarabine-based high-dose sequential chemotherapy (R-HDS) and autograft schedule as the first-line therapy [6]. The study aimed to determine the long-term efficacy and late clinical complications associated with an intensive chemo-immunotherapy program.

The present study included virtually all patients with MCL treated in Italy with the R-HDS protocol from April 1992 to May 2017. The study was performed in accordance with the Declaration of Helsinki and approved by the local Institutional Review Board. As detailed in the Supplementary Fig. S-1, 205 patients were included in the analysis and 199 were considered evaluable. Their main clinical features included: median age 58 years (23–65), 164 (82%) were male, 194 (97%) had Ann-Arbor stage III–IV, with 165 (83%) having Bone Marrow (BM) involvement and 50 (25%) B symptoms; 67 patients (34%) had high LDH levels; among 174 patients assessable for MIPI score, 69 (40%) showed intermediate-high/high risk score; there were no patients with CNS involvement. All patients had a biopsy proved diagnosis of CD20-positive MCL. Expert pathologists at each participating institution reviewed the histological samples in 182 out of 199 cases, confirming the initial diagnosis based on the 2017 WHO Classification [7]. At histological review, a typical cytomorphological pattern was observed in 156 patients, and a blastoid variant was observed in the remaining 26 patients (14%). The Ki-67 staining was assessed on 96 samples, 59 (61%) showed <30% values, while 37 (39%) had values of 30% or above.

The treatment program was described previously and is illustrated in Supplementary Fig. S-2 [6]. Besides 186 patients treated with the standard R-HDS program, five patients received high-dose methotrexate (8 g/m2, i.v.), in place of hd-Ara-C, and 13 patients (6.5%), treated before 2000, received the HDS regimen without rituximab. Peripheral blood stem cells (PBSC) harvest was scheduled at haematological recovery following hd-cyclophosphamide, a second PBSC harvest was performed after the hd-Ara-C, in case of inadequate previous harvest, or when there was initial BM involvement. The program included a first ASCT, followed when feasible by a second ASCT, as detailed in Fig. S-2. ASCT was not performed in 17 patients (8.5%), due to disease progression or toxic events (see Fig. S-1). Of the remaining 182 patients (91.4%), 95 (48%) underwent both ASCT procedures. The reasons that patients did not proceed to the second planned ASCT are detailed in Fig. S-1. Patients with initial bulky or residual disease after the ASCT received involved field radiotherapy within 2–3 months after the ASCT. No patient received maintenance Rituximab following R-HDS.

At the end of the program, 170 patients (85%) achieved either a CR or unconfirmed CR (CRu), 14 (7%) achieved a partial response, and 11 (6%) showed progressive or stable disease, while four patients (2%) died for early treatment-related deaths (see below causes of death). As of June 2019, with a median follow-up of 7 years (range: 0.5–25 years), 119 patients were alive (60%), 96 patients were in a continuous first CR (1st CCR), 20 patients were in >1st CR, and 3 patients were alive with active disease. Among the 96 patients in long-lasting remission, 80 (83%) remained in 1st CCR for more than 5 years.

**Fig. 1:**
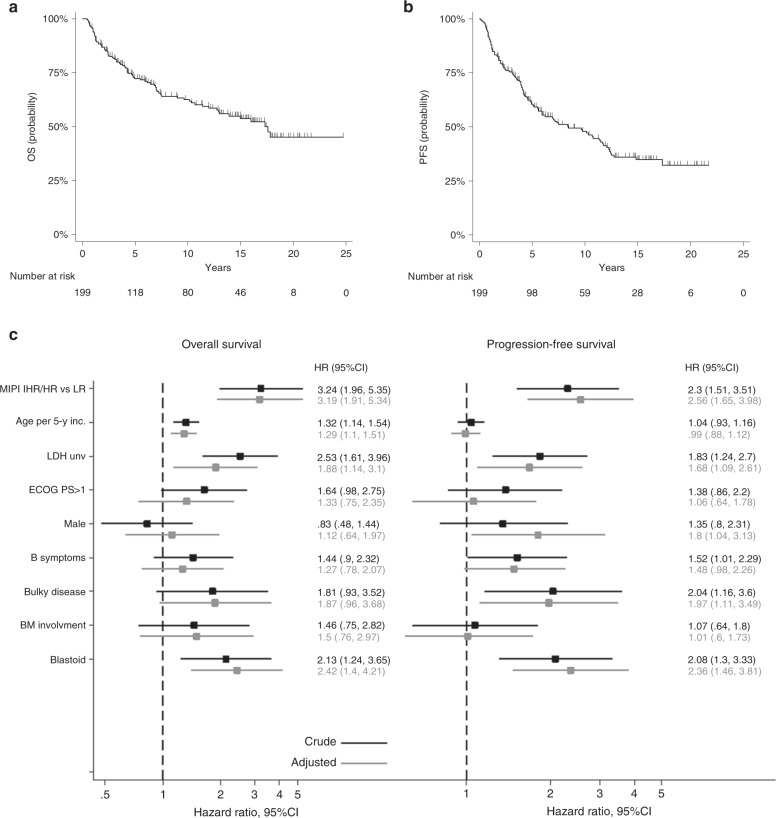
Long-term outcome and main prognostic factors in 199 MCL patients receiving R-HDS. **A** Overall Survival (OS) curve. At a median follow-up of 7 years, the 5 and 10-year OS rates were 72.3% (95% CI : 65.2–78.2), and 62.5% (95% CI: 55–69.4), respectively. **B** Progression-free survival (PFS) curve. At a median follow-up of 7 years, the 5 and 10-year PFS rates were 60.5% (95% CI: 53–67.1), and 48% (95% CI: 40.1–55.3), respectively. **C** Forest plots displaying factors that influenced long-term outcome. MCL: mantle cell lymphoma, LR, IHR/HR: low-risk, intermediate-high risk/high-risk MIPI scores, MIPI: MCL international prognostic index, LDH unv: lactate dehydrogenase upper normal value, ECOG PS: Eastern Cooperative Oncology Group performance status, BM: bone marrow. In the multivariable analysis, the MIPI score and its components (age, ECOG PS, and LDH) were not adjusted for one another.

According to an intent-to-treat analysis, the 7-year OS was 67% (95% CI: 59.4–73.3, Fig. 1A). There were no significant differences in the 7-year survival projections among patients treated before 2.000 (*n* = 58, 7-y OS = 63.1%), those treated in the period 2000–2010 (*n* = 90, 7-y OS = 65.1%) and those treated after 2010 (*n* = 51, 7-y OS = 80.2%) (logrank test *p* = 0.2995). The OS rates were significantly different among patients with low-risk MIPI score compared to those with intermediate-high and high-risk scores (see Supplementary Fig. S-3, A). After a median follow-up of 7 years, the 7-year PFS was 53.2% (95% CI: 45.5–60.3, Fig. 1B). The PFS rates were significantly different between those with low MIPI scores and those with intermediate-high or high risk scores (Fig. S-3, B). The DFS at 7 years for the entire cohort was 60.2% (95% CI: 51.5–68; Fig. S-3, C). Again, the low MIPI group had significantly different DFS rates than the intermediate-high and high-risk MIPI group (Fig. S-3, D).

As shown in Fig. 1C, a multivariable analysis showed that significantly lower survival rates were associated with an intermediate-high or high MIPI score vs. a low MIPI score, older age, high lactate dehydrogenase, and a blastoid histology. A significantly worse PFS was associated with an intermediate-high or high MIPI, high lactate dehydrogenase, bulky disease, and a blastoid histology (see Fig. 1C). There were no statistically significant differences between patients receiving one ASCT vs. those receiving two ASCT, both in terms of OS (not shown) and PFS (Supplementary Fig. S-4, A). Among the 26 patients with a blastoid histology, 11 survived (42%), at a median follow-up of 13.7 years (range 4–17 years), and 15 (58%) died, with a 13-year OS of 37.5%. Both OS and PFS curves were significantly better in patients with typical vs. blastoid histology (see Fig. S-4,B showing PFS curves). In general, the intensive treatment with autograft did not abrogate the predictive value of main prognostic factors identified in MCL, with the exception of the proliferative index. Indeed, there were no significant differences both in terms of OS and PFS between patients with Ki-67 < or = />30%.

Overall, 80 patients died (40%), four of them (2%) within 100 days from enrolment (three sepsis and one cardiac arrhythmia). Twenty-eight patients (14%), including the four early fatal cases, died of non-disease related causes in their first CR due to secondary malignancies (*N* = 19; 9.5%), infections (*N* = 5; 2.5%), cardiac failure (*N* = 3; 1.5%), and suicide (*N* = 1); four more patients died during >1st CR, after salvage treatment. Disease progression was the cause of death in 48 patients (24%). Twenty-nine patients (14.6%) developed a secondary malignant neoplasm, with 18 (9%) solid cancers and 11 (5.5%) haematological malignancies; secondary malignancy had a fatal outcome in 20 cases.

The results demonstrated that intensive, R-HDS chemo-immunotherapy had potent anti-lymphoma efficacy against MCL, both at short- and long-term, with survival results that compare favorably with other rituximab supplemented high dose chemotherapy programs. Our long-term analysis showed that nearly half of the patients maintained persistent clinical remission at several years after treatment completion. Some of these patients with long-lasting CR might have been cured of their disease. Thus, the results raised the issue of whether MCL is curable, whereas currently its eradication is considered impossible. Moreover, most patients experienced a prolonged interval to the next treatment, which allowed the administration of effective salvage treatments, in cases of disease recurrence. Thus, when we combined long-term outcomes of patients in the 1st CR and subsequent CRs, the OS was definitely satisfactory for the entire series of patients treated with R-HDS with ~70% of patients alive after a median of 7 years. These findings confirmed the marked improvement in life expectancy for patients with MCL during the last decade [8–10].

A significant matter of concern was the overall treatment toxicity. The acute treatment-related mortality of 2% was comparable to that reported by other intensive approaches [11]. In addition, at long-term we found 28 deaths (14%) among patients in their 1st CR, mainly due to secondary malignancies, infections, and cardiac toxicities. The late toxicities might even further increase as the years passed. Future long-term follow-up studies on clinical trials based on innovative drugs will be important to determine whether the combined use of innovative drugs and chemotherapy might hold promise by increasing disease control with manageable toxicity. In this view, it is of relevance the recent report of a prospective trial by the FIL cooperative study group indicating that Lenalidomide maintenance after an induction with a RHDS-like scheme and ASCT consolidation prolongs PFS in MCL patients. [12]

In summary, the data here presented could serve as a solid benchmark for newer approaches. Indeed, the results indicated that adults with MCL may have a long life expectancy, with a high proportion of patients surviving long-term with no evidence of disease.

## Supplementary information

Supplementary Figures

LEGENDS TO SUPPLEMENTARY FIGURES
