# Supplementary material for: Upfront intensive chemo-immunotherapy with autograft in 199 adult mantle cell lymphoma patients: prolonged survival and cure potentiality at long term
Source: Bone Marrow Transplant. 2021 Jul 7;56(10):2606–9. doi: 10.1038/s41409-021-01391-x (PMC8486659; doi:10.1038/s41409-021-01391-x)
Supplement: Supplementary file 1 — Supplementary Figures [file 41409_2021_1391_MOESM1_ESM.docx]

**Supplementary Figure S-1**

**
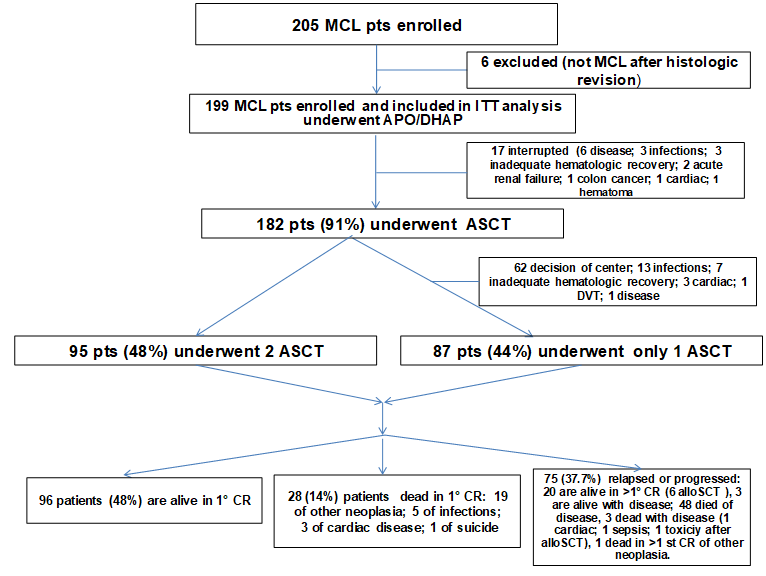
**

**Supplementary Figure S-2**


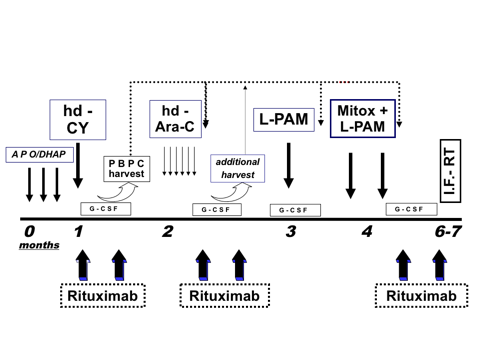


ALIVE: 119 patients
1^st^ CR: 96 patients
>1^st^ CR: 20 patients
Alive with disease: 3 patients

**Supplementary Figure S-3**

**A**

**B**

**D**

**C**

**Supplementary Figure S-4**

**A**

**B**
