# Supplementary material for: Upfront intensive chemo-immunotherapy with autograft in 199 adult mantle cell lymphoma patients: prolonged survival and cure potentiality at long term
Source: Bone Marrow Transplant. 2021 Jul 7;56(10):2606–9. doi: 10.1038/s41409-021-01391-x (PMC8486659; doi:10.1038/s41409-021-01391-x)
Supplement: Supplementary file 2 — LEGENDS TO SUPPLEMENTARY FIGURES [file 41409_2021_1391_MOESM2_ESM.docx]

**LEGENDS TO SUPPLEMENTARY FIGURES:**

**Figure S-1.** CONSORT diagram showing the selection, treatment allocation, and analysis of patients with Mantle-cell Lymphoma (MCL) receiving R-HDS during the period April 1992 to May 2017 in Italy. APO: doxorubicin, vincristine, and prednisone; DHAP: dexamethasone, cisplatin, and cytarabine; pts: patients; ASCT: autologous stem cell transplantation; DVT: deep venous thrombosis; ITT: intention-to-treat

**Figure S-2.** Schematic illustration of the R-HDS scheme. APO, doxorubicin 50 mg/m^2^ i.v., day 1; vincristine 1.4/m^2^ i.v., day 1; prednisone 40 mg/m^2^ oral, days 1-21; DHAP, dexamethasone 40 mg i.v., days 1-4; cisplatin 100 mg/m^2^ i.v., day 1; Ara-C 2 g/ m^2^ i.v., b.i.d., day 2; hd-CY, high dose cyclophosphamide, 7 g/m^2^ i.v. day 1; hd-ARA-C, high-dose cytarabine, cytarabine 2 g/m^2^ i.v. b.i.d., days 1-6; hd-L-PAM (autograft 1): melphalan 180 mg/m^2^ i.v., day 1; mitoxantrone 60 mg/m^2^ i.v., day -5+L-PAM, melphalan 180 mg/m^2^ i.v., day -2 (autograft 2); in a subset of cases autograft 2 was performed with BEAM, carmustine 300 mg/m^2^ i.v., day -6, etoposide 200 mg/m^2^ , days -5 to -2, cytarabine 200 mg/m^2^ i.v. b.i.d., days -5 to - 2, melphalan 140 mg/m^2^ i.v., day-1; Rituximab, given i.v. at 375 mg/m^2^; G-CSF, granulocyte colony-stimulating factor; PBPC, peripheral blood progenitor cells.

**Figure S-3.** Long-term outcome of 199 R-HDS treated MCL patients according to the MIPI score.

**(A)** Overall Survival (OS) of patients with Low Risk (LR, solid line) or Intermediate-High Risk/High Risk (IHR/HR, dashed line) MIPI (MCL international prognostic index) scores. The 5 and 10-year OS rates are respectively: 86% (95% CI: 77.1 to 91.4) and 81% (95% CI: 71 to 87.4) among patients with low-risk MIPI score; 56% (95% CI: 43 to 67.2) and 40.2% (95% CI: 27 to 53.1) among those with intermediate-high- and high-risk MIPI scores (log-rank test p<0.001 between the two patient groups).

**(B)** Progression-free Survival (PFS) of patients with Low Risk (LR, solid line) or Intermediate-High Risk/High Risk (IHR/HR, dashed line) MIPI (MCL international prognostic index) scores. The 5 and 10 year PFS rates were, respectively: 76% (95% CI: 66 to 83.1) and 65.4% (95% CI: 54.5 to 74.4) for the low MIPI group; 45% (95% CI: 32.4 to 57) and 31.1% (95% CI: 19.1 to 44) for the intermediate-high and high MIPI group. (log-rank test p<0.001 between the two patient groups).

**(C)** Disease-free Survival (DFS) Curve. At a median follow-up of 5 years, the 5 and 10-year DFS rates were 66.3 (95% CI 58 to 73.3) and 54.2 (95% CI 45.2 to 62.4), respectively.

**(D)** Disease-free Survival (DFS) of patients with Low Risk (LR, solid line) or Intermediate-High Risk/High Risk (IHR/HR, dashed line) MIPI (MCL international prognostic index) scores. The 5 and 10-year DFS rates were, respectively: 80% (95% CI:69.4 to 87) and 67% (95% CI: 55 to 76.5) for the low MIPI group; 53% (95% CI:38 to 65.6) and 39.6% (95% CI: 24.2 to 55) for the intermediate-high and high MIPI group. (log-rank test p<0.001 between the two patient groups).

**Figure S-4.** **Progression-free Survival according to Autograft and histology**

**(A)** Progression-free survival (PFS) of patients receiving one (solid line) or two (dashed line) autograft: There were no statistically significant differences between the two groups of patients in terms of PFS (HR 1.10 95% CI 0.70-1.71,p=0.682).

**(B)** Progression-free survival (PFS) of patients with typical histology and those with blastoid variant. The 5 and 10-year PFS rates are respectively: 64.0%% (95% CI: 55.5 to 71.3) and 48.8% (95% CI:39.6 to 57.4) among patients with typical histology; 39.9% (95%CI: 20.9-58.4) and 31.1%(95% CI: 14.2 to 49.7) among those with blastoid variant (log-rank test p=0.004 between the two patients groups).
